# The In Vivo, In Vitro and In Ovo Evaluation of Quantum Dots in Wound Healing: A Review

**DOI:** 10.3390/polym13020191

**Published:** 2021-01-07

**Authors:** Atiqah Salleh, Mh Busra Fauzi

**Affiliations:** Centre for Tissue Engineering and Regenerative Medicine, Faculty of Medicine, Universiti Kebangsaan Malaysia, Cheras, Kuala Lumpur 56000, Malaysia; atqhsalleh@gmail.com

**Keywords:** quantum dots, nanotechnology, wound healing, antibacterial properties, angiogenesis, rapid wound contraction

## Abstract

Wound is defined as primarily damaged or disruption of skin contributed to the loss of its microstructure stability and which undergoes complex wound healing process. However, there are tons of factors that could affect the wound healing process such as infection and slow angiogenesis. Involvement of nanotechnologies therapies in wound care research aims to facilitates this healing process. Quantum dots (QDs) are an advanced nanomaterial technology found to be useful in clinical and biomedical applications. This review has been carried out to provide a summary of the application of QDs in acute or chronic wound healing. A thorough searching was done via Web of Science and SCOPUS database to obtain relevant articles including the in vivo, in vitro and in ovo studies. The results demonstrated a similar effect of different types of QDs, or an improvement of QDs in wound healing, antibacterial and angiogenesis properties. This review demonstrated the effectiveness of QDs for the wound healing process mainly by their antibacterial activity. Uniquely, the antibacterial effect unraveled an increasing trend over time influenced by the various concentration of QDs. In conclusion, the application of QDs support the wound healing phases and proven to be effective in vivo, in vitro and in ovo. However, the future QDs work should focus on the molecular level for the details of cellular interactions and pathways.

## 1. Introduction

Skin is the largest organ of the body which acts as a front-liner of defense mechanism [1]. It is vital to keep the skin integrity and stability for normal function in balancing the body homeostasis. Furthermore, skin plays the main role as a barrier to protect the body against infection, fluid imbalance and thermal dysregulation [2]. The chances of survival will be decreased upon the compromised barrier especially when open injuries are involved. The skin wound is defined as damaged or disruption towards the skin epidermis (ruptured epithelium continuity) and dermis (matrix loss or discontinuity) due to possible various factors and causes including burns, cuts, traumatic injuries, post-surgical injuries, cancers, chemical injuries and ulcers [3].

**Figure 1:**
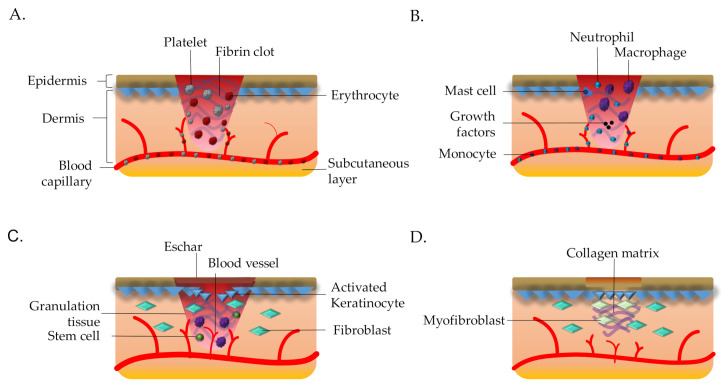
The myriad of wound healing processes. (**A**) Hemostasis process involves excretion of platelet to reduce the blood loss and formation of preliminary matrix (fibrin clot). (**B**) Inflammation involves the inflammatory cells to fight infection and debris removal. (**C**) Proliferation process where keratinocytes migrate to reduce the wound gap, fibroblasts proliferation replaces initial fibrin clot with granulation tissue and formation of new blood vessels occurs. (**D**) Remodeling phase causes the wound contraction that initiated by fibroblasts and myofibroblasts. Adapted with permission [8]. Licensed under CC BY 4.0.

There are four stages of skin wound: superficial which involve the epidermal layer only (Stage 1), partial-thickness skin loss which affects the epidermis and dermis (Stage 2), full-thickness skin loss extends into the dermis and affects the adipose tissues (Stage 3) and full-thickness skin loss which also extend through the dermis and adipose tissues that expose the muscle or bone (Stage 4) [4,5]. Figure 1 shows the overall wound healing process in normal condition. Besides, the wound can characterize as acute or chronic depends on the duration of the healing process. If the wound able to progress through all stages of wound healing more than 6 h but less than five days, it is classified as acute [6]. On the other hand, when the healing process still incomplete after three months of injury, it is classified as chronic wound [7]. Therefore it is crucial to understand skin wound healing because of its complicated and dynamic process.

Rapid wound closure and regeneration of the damaged skin is essential to maintain and re-develop the skin integrity [9]. Re-establishment of skin integrity and its native functions must go through a highly regulated process involving various types of cells and mediators under dynamic phases post-wounding. In a normal human body, the wound healing process is usually characterized as four sequential and overlapping dynamic phases including homeostasis, inflammation proliferation, and remodeling [2,10,11]. The details of biological events for each wound healing phases are simplified under Table 1. The duration of the wound healing depends on the degree of the wound itself such as stage 1, stage 2 and stage 3 or 4 wounds may take a few days and more than a month, respectively. Although the skin microenvironment is restored, the scar only can generate 30% less mechanical strength than healthy tissue [7]. The complication in any of the abovementioned steps may result in impaired wound healing especially chronic hard-to-heal ulcers, which presents as a major and increasing burden to our society including health and economic with a high increment of skin injury and hospitalization cost for wound managements [12,13,14], respectively. The application of tissue engineering able to help with regeneration of tissue by providing suitable microenvironment to support cell growth [15].

There are also other factors that may influence the healing process such as oxygenation, stress and other related-diseases [10]. Lack of oxygen can cause chain of negative side effects which could worsen the wound e.g., the vascular complication. The vascular complication occurs when there is limitation in delivering oxygen rich blood towards the wound site that needed for angiogenesis which then leads to other complications such as wound hypoxia. Wound hypoxia takes place when there are insufficient nutrients or oxygen delivery needed to regenerate tissue [19]. Patients suffer from diabetes also shows dearth of wound healing capability due to perturbation in vascular integrity. Diabetics wound exhibit insufficient angiogenesis needed for wound repair due to decrease in vascularity as well as vascular integrity [20]. Psychological stress can also induce poor wound healing properties as our body activates hormones which directly influence several components of wound healing process e.g., glucocorticoids. Overexpression of glucocorticoids, which is known as strong anti-inflammatory agents, affects the inflammatory stage of wound healing [21,22].

However, the topmost common factors affecting wound healing is a secondary bacterial infection. One of the most efficient way to fight against the growth of bacteria is exposure towards antibacterial metals such as zinc, silver, titanium etc. Nevertheless, the uses of these large quantity of these metals could have negative impacts on human health and the environment. Therefore, the development of nanoparticles become the best solution to decode the problems arise in the uses of heavy metals as well as increases the efficiency of the metal towards the antibacterial activity. Recent technological advancement has improved the biomedical fields especially with the development of nanotechnologies.

### 1.1. Nanotechnologies

**Figure 2:**
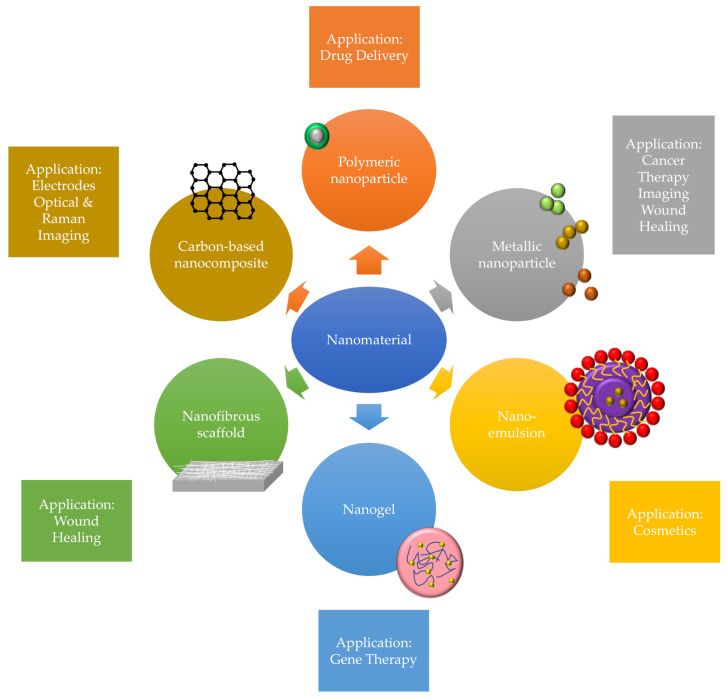
The types and application of nanomaterials.

The development of nanotechnologies provided an improvement in health industries with current innovation of functional and smarter nanomaterials to tackle the drawbacks for unintended wound as well as provide better outcomes among patients. Recently, the nano-based technologies have shown significant roles in wound healing and regenerative medicine applications. Nanotechnologies also improved the interaction between tissue and materials through biomimetic approach, helps in tissue regeneration whilst provide better micro-morphology and properties resembling the native tissues [23]. There are different types of nanomaterials have been implemented in tissue engineering models such as the uses of metallic nanoparticles, polymeric nanoparticles, nano-emulsions, solid lipid nanoparticles, nanospheres, nanogels, nanofibrous scaffold and carbon-based nanocomposite that have shown positive result in wound healing research [24,25]. Figure 2 showed some example of nanomaterials used in wound healing. Nanoparticles primarily can be classified into two groups which are organic and inorganic nanomaterials.

Organic nanomaterials are fabricated through organic compound e.g., chitosan, curcumin, etc. The main objective for organic nanomaterials in wound healing application is to carry out the treatment or active ingredients to targeted cells [26]. Nanocapsules are known for their ability to enclose the active substances within their structures and release these products in specific time and place, which ensure more effective delivery. Nano-emulsions are also frequently used as drug carrier as they are easily incorporate biologically active ingredient [27]. For instance, curcumin nano-emulsion (Cur-NE) have portrayed anti-inflammatory properties which are crucial for wound healing [28]. The development of biodegradable nanotechnologies also have increased the usage of nanomaterials in clinical application and encourages the molecular interaction between cells and biomaterials [29].

Meanwhile, the inorganic nanomaterials are fabricated substances through inorganic compound such as polymer and metallic component. Metallic nanoparticles including silver, zinc, and gold exhibits unique physicochemical properties that excellent in accelerating wound healing process. In addition, silver nanoparticles have been used in wound dressings as they display oligodynamic effects towards bacteria [30]. Fabrication of nanofibrous scaffold could achieve through electrospinning methods and have been intensively used as cartilage tissue engineering product where it proven to enhance the chondrogenesis of stem cells [31]. The production of quantum dots (QDs) has elevated the nanoscience into higher strata due to its advanced involvement in various industries as well as with higher biocompatibility compared to metallic nanoparticles. 

### 1.2. Quantum Dots

QDs were first discovered by Ekimov and Onushenko in 1981, and are nanoscale semiconductor crystals and first nanotechnologies to be applied in biological science made of heavy metals [32,33]. QDs have shown great potential in several biomedical types of research including fluorescence imaging, disease detection, fluorescence assays for single protein track, drug discovery and intracellular reporting due to their mechanical and physicochemical properties [34]. QDs have five distinct properties that ameliorate tremendous research interest including (1) the nano size ranges from 4 until 12 nm in diameter, (2) narrow and size-tunable Gaussian emission spectra which excite to the near-infrared (NIR); lower than 650 nm, (3) self-luminescence due to their absorption extinction coefficients and high fluorescence quantum yields, (4) QDs are photochemically robust due to its inorganic composition and the fluorescence intermittency with observation of single dot event, (5) observing a single protein compound [35]. 

The synthesis of QDs results in organic capping ligands that make them biocompatible, and a biological targeting development which achieved by surface modification and linking with antibodies, peptides or small molecules [35,36]. QDs also have been applied in biomedical applications such as delivery of drug, bio-sensing and also tissue engineering [37]. QDs are ideal nano-carriers for the drug due to their high surface area to interact with other molecules especially their strong interactions with organic molecules and specific compounds [38]. Application of QDs in drug delivery has increased drug stability, prolonged in vivo circulation time, improve the distribution and metabolism process of drugs and enhance absorption [34]. The optical properties of QDs have been used for bio-imaging applications in various biological research for deep-tissue imaging with reduced light scattering and low tissue absorption [39]. Researchers worldwide used the QDs as fluorescence labeling for both in vivo cellular imaging and in vitro assay detection due to photoluminescence properties [40]. 

There have been increasing research on QDs in wound healing as these nanoparticles equip the same properties as their bulk counterparts and more stable due to their large surface area [41]. The unique physicochemical characteristics of QDs are highly beneficial in tissue engineering applications especially their antibacterial properties as these aspects are important in development of biomaterials. QDs also can enhance the mechanical strength of tissue scaffolds and hydrogels for wound healing, or for regenerative medicine [42]. Due to its nano-scale size (less than 20 nm), QDs have low toxicity towards the cells and have enzymatic functions such as oxidase which make it suitable to be incorporated into a bioscaffold [43]. The large surface area of QDs could bind to the ligands which involved in wound healing process. For example, carbon quantum dots proven to involve in angiogenesis process as these nanoparticles able to enhance the anti-angiogenic factors expression which is crucial to avoid overexpression of pro-angiogenic factors expression [44]. QDs also involve in signaling pathway which enhance the inflammation phase of wound healing as it increases the interleukin-6 expression [45]. Therefore, the myriad application of QDs in wound healing research have been chosen and discussed in this review.

In this review, a literature search was done through electronic databases was carried out to identify the in vivo, in vitro and in ovo study performed on the application of QDs in chronic and acute wound healing. 

## 2. Literature Search

**Figure 3:**
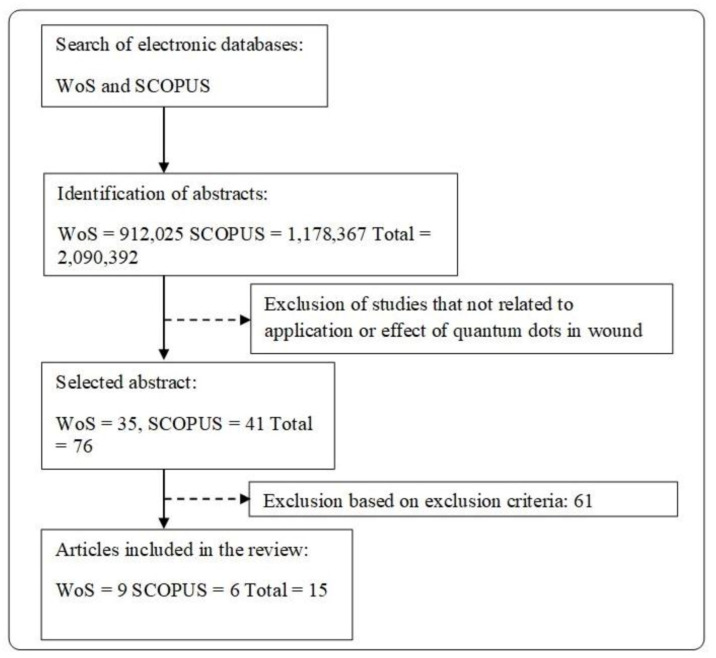
PRISMA flow diagram.

Literature search was done on the reports regarding the effect of quantum dots (QDs) on biomedical and clinical applications, especially in the wound healing process. Figure 3 shows the overall literature search of this review. Original research articles (in vitro, in vivo or in ovo study) which discuss the effects of QDs in wound healing (cell proliferation, cell migration, angiogenesis, and cell toxicity) and the main priority of QDs applications in tissue engineering that involved in wound care management. The studies involve a different type of QDs (derived from a different type of heavy metals) are included in this review. Studies on the cells involved during wound healing such as (1) fibroblasts; (2) keratinocytes; or (3) endothelial cells were also included. The exclusion criteria for this review would be all secondary literature and any original articles that have been wrote and submitted in other languages other than English. Studies focusing on the effect of QDs towards diseases e.g., cancer were excluded from the review. Figure 3 shows the flow diagram of articles selection.

## 3. Wound Healing Properties of QDs

The outcomes from the review highlighted the advantageous of quantum dots (QDs) in wound healing. There are various reports on the application of QDs in wound healing and it has been tested in vivo, in vitro and in ovo model. The overall result of QDs seems to be beneficial towards wound healing as the treatment group obtains better results compared to the control group. However, the combination of QDs with polymer e.g., carbon quantum dots with hybrid tannic acid and keratin (CQDs-TA/KA) hydrogel yields a more appealing result compared to QDs itself [46]. 

### 3.1. Wound Closure

The *in vivo* studies were used to study the biological effect of QDs in a dynamic and complex biosystem e.g., the wound closure. Apart from having QDs derived from various types of heavy metals, the papers were primary aim to assess their biological effect in selected wound model in each study. Most of the studies inflict a cutaneous incisional wound and there only one study that inflicts burn wound on the animal. The wound healing assessments were performed by gross morphology of the wound, the size of wounds and the histological evaluation using hematoxylin and eosin (H & E) staining to determine the condition of the regenerated tissue [47]. The in vivo studies reporting the effects of QDs on wound healing were simplified in Table 2.

Based on the results obtained, the QDs supports the wound healing process in vivo. The histological assessment shows the regeneration of tissue and formation of blood vessel when treated with QDs. Xiang et al., 2019 show even disposition of collagen and dense collagen fibers when treated with QDs [52]. Haghshenas et al., 2019 show that QDs involve the formation or regeneration of tissue at the wound site are faster in treated compare with non-treated model [51]. In the context of wound healing, progression in the inflammatory stage depends on the suitable microenvironment at the wound site. Infection is also one of the factors which affect the healing process where it is important for the removal of micro-organisms before advancing to the next stage of wound healing process [58]. Microbial infections may prolong the inflammatory stage and causing high expression of pro-inflammatory cytokines (interleukin 1 alpha, interleukin 1 beta and tumor necrosis factor alpha) that not only harmful towards infected cells but also healthy cells [59].

### 3.2. Antibacterial Effects 

The elevated prevalence of microbial infection increases the complication in wound healing especially in skin injuries. Thus, a higher rate of morbidity in conventional therapeutics creates a demand for alternative antibacterial agents to treat microbial infections such as QDs, silver and others. Briefly, the common antibacterial mechanism of QDs takes place via three molecular mechanisms: (1) disruption of cell walls or cells membrane, (2) production of reactive oxygen species (ROS) and (3) binding with nuclei materials such as DNA or RNA to inhibit the cell proliferation. The most common bacteria used in the antibacterial studies were *Escherichia coli* (*E. coli*; Gram-negative) and *Staphylococcus aureus* (*S. aureus*; Gram-positive). Generally, the antibacterial properties of QDs were analyzed using a plate counting method to calculate the colony-forming unit (CFU) of remaining bacteria after exposure to the particular treatment modalities and morphological studies on bacteria were conducted to compare the bacterial structure pre- and post-treatment. When the bacteria were exposed to QDs, the microbial membrane disrupt due to the high production of reactive oxygen species (ROS) which triggers significant lipid peroxidation on membrane [57]. The combination of QDs with polymer deems to have high antibacterial properties towards micro-organisms e.g., *S. aureus* even inhibits the growth of different drug-resistant bacteria e.g., Methicillin-resistant Staphylococcus aureus (MRSA) [60].

This review has stated that the antibacterial properties of QDs have a strong reaction towards both gram-negative and gram-positive bacteria [60]. However, antibacterial properties of QDs are proven higher towards gram-positive bacteria in comparison with gram-negative bacteria. There are eight antibacterial studies show that there is slight decrease of antibacterial properties of QDs for gram-studies show that there is a slight decrease in antibacterial properties of QDs for gram-negative bacteria than gram-positive bacteria. Malmir et al., 2020 have proven that a combination of carbon quantum dot with titanium oxide has a smaller bacterial inhibition zone in *E. coli* than with *S. aureus* [61]. The main reason is the complexity of the cell membrane in the gram-negative which consists of lipids, proteins and lipopolysaccharides that protect the gram-negative bacteria from bactericide [62,63]. QDs tend to have higher antimicrobial properties when they are in an excited state or exposed to light radiation as an example Liang et al., 2019 have tested zinc oxide quantum dots in both excited and normal state. The result was antibacterial rates and production of reactive oxygen species (ROS) were significantly higher in an excited state than normal state [57]. The photoactivated QDs produce more radicals that cause accumulation of ROS inside the cells which inhibit respiration and replication of microbes [64]. Table 3 shows the overall antibacterial activity exhibited by QD.

### 3.3. Angiogenesis

Angiogenesis is one of the important factors in wound repair which function to deliver the gaseous (e.g., oxygen) and nutrients at wound sites to further support the cell growth and tissue regeneration [65]. Angiogenesis process is majorly controlled by vascular endothelial growth factor (VEGF) expression which is a 35–45 kDa homodimer glycoprotein that stimulate the growth of endothelial cells and formation of new capillary tubes [66,67]. The angiogenesis properties of QDs can be evaluated by using the human umbilical vein endothelial cells (HUVECs) as angiogenic parameter in research model. HUVECs provide an in vitro model to evaluate the physiological and pathological processes of vascularization. The angiogenesis properties of QDs determined by the response of endothelial cells towards QDs. HUVECs regularly expressed the VEGF that a vital protein in angiogenesis [68]. Besides, the previous study demonstrated an in ovo approach also can be used for further understanding of angiogenesis properties. 

There are three articles on the angiogenesis properties of QDs [56,69,70]. Zhu et al., 2019 have conducted a study on retinal artilleries in vivo using rats [69]. The rats were treated with selenium quantum dots (SeQDs) and the artilleries were analyzed quantitatively using ultrasound. The result of the studies was measured by using MTT assay (HUVECs proliferation), in vitro Matrigel (formation of blood vessels) and protein expression (expression of proangiogenic protein). This research also performed in ovo angiogenesis assay where the angiogenic potential of CD-urea was assessed by using the carcinoembryonic antigen (CEA) assay. While Li et al., 2020 conduct research on carbon quantum dots derived from lysine and arginine [56]. The study used live and dead assay on HUVECs and had shown positive results in the treated group. The studies on angiogenesis properties of QDs have been summarized in Table 4. 

Angiogenesis occurs with the migration and mitogenic activation of the endothelial cells in the extracellular matrix of the wound bed. This results in a temporary increase of blood vessels at the wound site and included in the granulation tissue formation [71]. The excellent wound irrigation at the edge of the injury site was found to speed up the wound healing process, as this allows sufficient nutrient and oxygen supply [11]. Endothelial cells digest and infiltrate the underlying vascular basement membrane at the start of the cycle of forming capillary sprouts, invade the extracellular matrix (ECM) stroma [72]. Thus, formation of tube-like structures that continue to grow, branch and build networks, driven from behind by endothelial cell proliferation and pulled from the front by chemotaxis [73]. 

Sharma et al., 2019 have been investigating the interaction of carbon dot (CD)-urea towards endothelial cells [70]. The result demonstrates that HUVECs are dose-dependent upon treatment with cytocompatibility and hemocompatible CD-urea, which increases with proliferative and improved proangiogenic responses. The studies were subsequently verified in the studies of chorioallantoic membrane (CAM) in ovo chick. In ovo CAM method is described as employed shell cultures of chick embryos as it provides a natural environment of growing blood vessels [74,75]. CAM assay has been widely used to study angiogenesis due to its highly vascularized nature, high reproducibility, cost-effectiveness and closed-system environment (longer half-life compared to animal model) [76]. The expression of proangiogenic factors also plays a vital role in assuring good angiogenesis response. 

## 4. Future Perspective

From this literature search, most of the studies on QDs found were related to its self-illuminate properties. Accordingly, the result of the QDs were limited to its application in bio-sensing and bio-imaging. However, in terms of wound healing application, there are limitation in the literature distribution. Consequently, the underlying cellular mechanisms of QDs are yet to be known. Hence, more studies should be properly performed such as the effect of QDs in cell mechanism and longer observation period to learn the side effect of QDs. The data collected in the review also described that different types of QDs have distinguished findings which can be improved by focusing on one type of QDs to have a better understanding of its biological properties. Various type of wound could also influence the efficacy of the QDs e.g., full-thickness skin loss have slower healing rate than an acute wound which cause the time period of wound healing to differ even treated with the similar type of QDs [77]. Another factor that could affect these studies is the difference in the size of QDs. The size plays an important role in the antibacterial properties as the smaller size of nanoparticles tends to be more toxic towards bacteria e.g., nano silver particle size can alter its antimicrobial activity [78]. Semiconductors properties of QDs can be used in the biomedical application where application of electrical charges through QDs able provide better and faster migration of cells in human body. Other than that, the uses of QDs as an electronic implant that easily embedded through injection. Data collected throughout this review shows that QDs assisted in the acceleration of the wound healing process by eliminating microbial infection and promote angiogenesis. The use of QDs together with advanced technology of biomaterials could synergistically expedite wound care management worldwide.

## 5. Conclusions

Studies included in this overview have shown the efficiency of the application of quantum dots (QDs; derived from a different type of sources) in biomedical applications due to their wound healing, antimicrobial, and angiogenesis properties. There are many articles that proved the effect of QDs in wound healing and had been tested in vivo, in vitro and in ovo models. In terms of wound management, the high antibacterial properties of QDs towards drug-resistant bacteria making them one of alternative antimicrobial therapy. QDs also can enhance angiogenesis in the injury site as it helps in the expression of angiogenic proteins. From our perspective, the in vivo studies of QDs provide more impactful data on the efficiency of these nanoparticles in wound healing research as these data shown the effects of QDs in reconstruction of tissue and closure of wound. The data shown in our review supports the inkling of QDs in wound healing process. Therefore, further studies are required to be done for further understand the applications of QDs in wound healing especially in the molecular levels as well as the details involvement of nanoparticles at different wound healing stages.

## Figures and Tables

**Table 1:** Wound healing process.

Phase	Biological Events
Hemostasis [16]	Exposure of collagen initiates intrinsic and extrinsic clotting cascades Thrombocytes aggregated and triggering the vasoconstriction Blot clot formation to act as a temporary wound matrix—assist in migration of cells Blood vessels dilated; thrombocytes and leukocytes migrated after 5 to 10 min of vasoconstriction Platelets degranulated—cytokines and growth factors released into the wound
Inflammation [11]	The increasing number of leukocytes in the wound area Expression of pro-inflammatory cytokines caused by transmigration of neutrophils through endothelial cells Pro-inflammatory cytokines promote the adhesion molecules expression e.g., intercellular adhesion molecule 1 (ICAM1), vascular cell adhesion molecule 1 (VCAM1) and selectin (SELE) The neutrophils will migrate against the chemokine gradients, where there are high concentration of chemokines in this case wound site Neutrophils performed phagocytosis and produce cytokines such as tumor necrosis factor (TNF-α), interleukin-1 (IL-1) and interleukin-6 (IL-6) to increase the inflammatory response Monocytes will migrate to wound site and differentiate into macrophages after 3 days injury—attract other inflammatory cells and produce prostaglandins
Proliferation [17]	The proliferation of vascular endothelial cells and fibroblasts due to secretion of growth factors by inflammatory cells Collagen secreted by fibroblasts to replace the fibrin matrix Differentiation of fibroblasts into myofibroblasts expressing actin—contraction and reduction of the wound area Healthy tissues and endothelial progenitors initiated the angiogenesis Formation of granulation tissue—the invasion of vascular endothelial cells and capillaries
Remodeling [18]	Collagen III replaced by collagen I Myofibroblasts attach to collagen for wound contraction and help decrease development of scar Angiogenic process diminished—wound blood flow declines and metabolic activity slows down until it stops

**Table 2:** In vivo study on wound healing.

No.	References	Experimental Model	Type of Quantum Dots	Outcome Measures	Results	Conclusion
1	Ma et al., 2019 [48]	Sprague-Dawley rats 6 weeks old Weight 200 g Excision wound (1.00 cm^2^)	VO_x_NDs	1. Morphology of wound 2. Histological evaluation (H & E stain)	H_2_O_2_/ VO_x_NDs have 60% decrease in wound area compared to control and rigid epidermal layer after 6 days therapy	H_2_O_2_/VO_x_NDs group have the greatest wound healing capacity among all tested group
2	Zhao et al., 2019 [49]	Sprague-Dawley rats Weight 250 ± 20 g Full thickness wound (1.80 cm^2^)	NCQDs	1. Wound morphology 2. Histological evaluation (H & E stain) 3. White blood count (blood slide)	Treatment with NCQDs have significantly higher healing rate where the wound area is 0.2% at the 14th day of treatment and lower white blood count which is 1 × 10^10^ L^−1^ indicate decrease of inflammation in wound area	NCQDs show effective treatment towards wound healing
3	Bankoti et al., 2017 [50]	Albino Wistar rats Weights 150–200 g Excision wound (3.14 cm^2^)	CND	1. Morphology evaluation 2. Histological examination (H & E stain)	Treatment of OCNDs had more than 80% of healing compare to control (65%) and shown to have intact dermal and epidermal structure which does not show signs of inflammation nor infection	Topical application of OCNDs improved the wound healing process
4	Haghshenas et al., 2019 [51]	Wistar rats Burn wound	GQDs	1. Morphology study of recovery process 2. Histological assessment (H & E stain and Masson’s trichrome staining)	Treatment group have higher healing rate than control group and formation of fibroblasts are 10% higher than control	GQDs able to accelerate the repair of skin lesion in burn wound healing model
5	Ren et al., 2020 [46]	Rats 10–12 weeks old Weight 250–300 g Full thickness wound (1.50 cm^2^)	GOQDs	1. Gross morphology of wound 2. Histological assessment (H & E stain)	Treatment with TA/KA-GOQDs show 98% of wound are closure and matured epidermal layer after 16 days of treatment	TA/KA-GOQDs proves its ability to treat wounds within short period of time and without side effects
6	Xiang et al., 2019 [52]	Rats Incision wound	CQDs	1. Gross morphology of wound 2. Histological assessment (H & E stain and Masson’s trichrome staining)	DFT-C/ZnO-hydrogel-treated group have 95.7% of wound closure by 10 days of treatment. H & E staining show that this treatment group have complete epidermal structure in 2 days Dense collagen fiber have been observed in treatment group after 10th day of treatment	Treatment with DFT-C/ZnO-hydrogel groups exhibit the best wound healing results
7	Omidi et al., 2017 [42]	Rat Weights 260 g Excision wound (1.00 cm^2^)	CND	1. Morphology evaluation	The wound heals at ~100% at 16th days in with CDs/chitosan nanocomposite compared to 40% of control group	The characteristic of CDs/chitosan shown to be beneficial as wound dressing products
8	Tian et al., 2019 [53]	BALB/c mice 8 weeks old Incision wound	MoS_2_QDs	1. Morphology evaluation	The infected wounds almost 90% completely healed in photoexcited MoS_2_QDs group, compared to control group	The potential application of the of MoS2 QDs was demonstrated great improvement of wound healing
9	Yin et al., 2016 [54]	Female BALB/c mice 8 weeks old Weight 18–23 g Excision wound (0.78 cm^2^)	MoS_2_NF	1. Gross morphology of wound 2. Histological assessment (H & E stain and Masson’s trichrome stain)	The treatment groups show formation of epidermal layer for wound closure at 5th day of treatment and attachment of collagen fiber with dermal layer	The MoS_2_NF shown improvement of wound healing in short period of time
10	Sun et al., 2014 [55]	Male Kunming mice 6–8 weeks old Weight 180–220 g Excision wound (0.04 cm^2^)	GQDs	1. Gross morphology of wound	Treatment with H_2_O_2_ and GQD band aid groups shows no significant results in wound closure	Treatment with GQD band aid groups as wound dressing shows no significant result for wound healing
11	Li et al., 2020 [56]	Male mice 6–8 weeks old Weight 180–220 g Incision wound (1.6 cm^2^)	CQDs	1. Gross morphology of wound 2. Histological assessment (H & E stain)	CQDs-treated group show complete closure of wound and higher degree of healing within 5 days of treatment	CDQs contribute to faster wound healing and great potential for wound dressing
12	Liang et al., 2019 [57]	Male mice Excision wound (0.79 cm^2^)	ZnOQDs	1. Morphology assessment	Treatment of ZnOQDs with GO-CS hydrogel shown 90% of wound closure after 14th day of treatment	ZnOQDs imbedded in GO-CS hydrogel show potential to be used for wound dressing

**Table 3:** Antibacterial properties of quantum dots.

No	References	Experimental Model	Type Of Quantum Dots	Outcome Measures	Results	Antibacterial Mechanism	Conclusion
1	Yin et al., 2016 [54]	1. Amp^r^ *E. coli* 2. *B. subtilis*	MoS_2_NF	1. Plate counting method 2. Morphology of the bacteria 3. Characterization of bacterial death	The bacteria that were incubated with MoS_2_ + H_2_O_2_ and exposed to the 808 nm laser show reduction in the bacteria viability and the bacteria inactivation of bacteria are 97% and 100% for Amp^r^ *E. coli* and *B. subtilis*, respectively	The nanoparticles bind to bacterial membrane and decrease the integrity of the membrane.	PEG-MoS_2_NFs possess peroxidase catalytic activity and show to be effective for antibacterial properties
2	Omidi et al., 2017 [42]	1. *Staphylococcus aureus*	CND	1. Disc diffusion method 2. Optical density	The CDs showed inhibition zone of 3.1 mm, 3.7 mm and 4.6 mm for 5%, 10% and 15% *v*/*v*, respectively and inhibition of *Staphylococcus aureus* in the concentration of CDs was more than 10 mg ml^−1^	No mechanism of action has been scrutinized in the research article.	The chitosan/CDs nanocomposites had antibacterial activity by inducing a clear inhibition of bacterial growth
3	Tian et al., 2019 [53]	1. *E. coli* 2. *S. aureus*	MoS_2_QDs	1. Plate counting method 2. Morphological observation of bacterial death 3. ROS measurement	The survival rates of bacteria were still above 80% for both *S. aureus* and *E. coli* treated with MoS_2_QDs	The production of reactive oxygen species (ROS)	Treatment of MoS_2_QDs may be effective towards Gram-positive but not in Gram-negative bacteria due to its dual layer of membrane
4	Xiang et al., 2019 [52]	1. *E. coli* 2. *S. aureus*	CQDs	1. Spread plate method 2. Morphology of bacteria	The antibacterial rates of the DFT-C/ZnO-hydrogel for *S. aureus* and *E. coli* are 78.9% and 70.7%, respectively and the cellular membrane are disrupted when exposed to treatment group in 15 min.	The release of Zn^2+^ ion into the bacterial membrane which increase the oxidative stress in bacteria.	The combination of carbon quantum dots/ZnO and folic acid-conjugated PDA hydrogel have shown high antibacterial properties
5	Liang et al., 2019 [57]	1. *E. coli* 2. *S. aureus*	ZnOQDs	1. Spread plate method 2. Gross appearance of bacteria 3. Characterization of bacterial death	The antibacterial efficacy was significantly improved by 98.90% against *S. aureus* and by 99.50% against *E. coli* when the ZnO QDs@GO-CS hydrogel was under 808 nm light irradiation	The release of Zn^2+^ which inhibit respiratory enzymes and ROS production.	The ZnO QDs@GO-CS hydrogel have a higher antibacterial property when expose to light radiation.
6	Sun et al., 2014 [55]	1. *E. coli* 2. *S. aureus*	GQDs	1. Disk diffusion assay 2. Growth-inhibition assay 3. Morphology of bacteria	Treatment with H_2_O_2_ with both GQDs cause the number of bacteria decreases and the bacterial surface became rough and wrinkled	The peroxide—like activity of GQD causes the loss in the integrity of cell wall and thus rupture the bacterial membrane.	The antibacterial ability of H_2_O_2_ had been remarkably improved with the help of GQDs
7	Zhao et al., 2019 [49]	1. *S. aureus* (ATCC6538, ATCC43300) 2. *S. epidermidis* 3. Methicillin- resistant *Staphylococcus aureus* 4. *E. coli* 5. *Salmonella paratyphi-β* 6. *Pseudomonas aeruginosa* 7. *Enterococcus faecalis*	N-CQDs	1. Disk diffusion method 2. Broth diffusion method 3. Characterization of bacterial death	There was 15.5 nm of inhibition zones observed on the agar plates that incubate *S. aureus* (ATCC6538 and ATCC43300), *S. epidermidis*, and *MRSA* and minimum inhibition concentration (MIC) results are measured 0.128 and 0.256 mg/mL on NCQDs on and *S. aureus* (ATCC6538)	The positively charged NCQD bind with negatively charged bacteria causing rupture on the cell membrane.	NCQDs exercised broad antimicrobial activity over various bacterial forms
8	Ren et al., 2020 [46]	1. *E. coli* 2. *S. aureus*	GOQDs	1. Spread plate method	The bacterial survival rate decreases to 20% and 30% for *S. aureus* and *E. coli* respectively in TA/KA-GOQDs	The antibacterial mechanism of nanoparticles does not explain in the article.	Treatment of GOQDs with TA/KA hydrogels have high antibacterial properties
9	Ma et al., 2020 [48]	1. *E. coli* 2. *MRSA*	Vanadium oxide nanodots	1. Spread plate method 2. Morphology studies on bacteria	The CFU of H_2_O_2_/VO_x_NDs are the lowest and the survival rate of bacteria also significantly the lowest, >20%, when compared to other treatment groups	The production of ROS which rupture the bacterial membrane.	H_2_O_2_/VO_x_NDs can inhibit growth of drug-resistant bacteria
10	Malmir et al., 2020 [61]	1. *E. coli* 2. *S. aureus*	CQDs	1. MIC test 2. Characterization of bacterial death	The CQD-TiO_2_ NPs inhibition zone was seen around *S. aureus* bacteria but have no effect on *E. coli*	The production of ROS,	The antibacterial activity of CQD-TiO_2_ NPs against *E. coli* was lower than *S. aureus*
11	Li et al., 2020 [56]	1. *E. coli* 2. *S. aureus*	CQDs	1. MIC test 2. Time-kill study 3. Bacterial morphology	The MIC values of CQDs and Arg-CQDs are 62.5 µg/mL and 125 µg/mL for *E. coli*, respectively, and 31.25 µg/mL and 62.5 µg/mL for *S. aureus*, respectively. The cellular membrane of the bacteria is disrupted when exposed to treatment.	The electrostatic interaction of positively charged nanoparticles and negatively charged bacteria disrupt the bacterial membrane and the release of ROS.	CQDs show high inhibitory activities for both *E. coli* and *S. aureus*

**Table 4:** Effect of quantum dots to angiogenesis.

No	References	Experimental Model	Type of Quantum Dots	Outcome Measures	Results	Conclusion
1	Li et al., 2020 [56]	HUVECs	CQDs	1. CCK-8 tests 2. Live/dead assay	Cell viability of HUVECs is more than 85% at a concentration of 250 µg/mL	CQDs effectively promote the growth of HUVECs with a high survival rate
2	Sharma et al., 2019 [70]	HUVECs	CQDs	1. Cell Proliferation assay 2. In vitro tube formation assay 3. In Ovo angiogenesis assay 4. qPCR	The development of capillary network in HUVECs model has been significantly increased compared to control as well as high expression of VEGF	The CD-urea showed a proangiogenic response in HUVECs model
3	Zhu et al., 2019 [69]	HUVECs	SeQDs	1. Quantitative analysis of arteries 2. Protein expression	The treatment group of A-SeQDs have the highest diameter of arteries formed and increase NOS activity and NO production	SeQDs proved to promote angiogenesis properties

## Data Availability

No new data were created or analyzed in this study. Data sharing is not applicable to this article.

## Abbreviations

QDs	Quantum dots
VACM1	Vascular cell adhesion molecule 1
SELE	Selectin E, endothelial adhesion molecule 1
TNF-α	Tumor necrosis factor alpha
IL-1	Interleukin-1
IL-6	Interleukin-6
MRSA	Methicillin-resistant Staphylococcus aureus
VEGF	Vascular endothelial growth factor
ROS	Reactive oxygen species
CFU	Colony-forming unit
HUVEC	Human umbilical vein endothelial cells
ECM	Extracellular matrix
MIC	Minimum inhibition concentration
